# A sciaenid swim bladder with long skinny fingers produces sound with an unusual frequency spectrum

**DOI:** 10.1038/s41598-020-75663-9

**Published:** 2020-10-29

**Authors:** Hin-Kiu Mok, Shih-Chia Wu, Soranuth Sirisuary, Michael L. Fine

**Affiliations:** 1 Department of Oceanography, National Sun Yat-Sen University, Lien-hai Rd., Kaohsiung, 80424 Taiwan, R.O.C.; 2 Department of Aquaculture, Faculty of Fisheries, Kasetsart University, 50 Ngam Wong Wan Rd., Ladyaow Chatuchak, Bangkok, 10900 Thailand; 3 Department of Biology, Virginia Commonwealth University, Richmond, VA 23284-2012 USA; 4 National Museum of Marine Biology and Aquarium, 2 Houwan Road, Checheng, Pingtung 944 Taiwan, R.O.C.

**Keywords:** Zoology, Ecology, Anatomy

## Abstract

Swim bladders in sciaenid fishes function in hearing in some and sound production in almost all species. Sciaenid swim bladders vary from simple carrot-shaped to two-chambered to possessing various diverticula. Diverticula that terminate close to the ears improve hearing. Other unusual diverticula heading in a caudal direction have not been studied. The fresh-water Asian species *Boesemania microlepis* has an unusual swim bladder with a slightly restricted anterior region and 6 long-slender caudally-directed diverticula bilaterally. We hypothesized that these diverticula modify sound spectra. Evening advertisement calls consist of a series of multicycle tonal pulses, but the fundamental frequency and first several harmonics are missing or attenuated, and peak frequencies are high, varying between < 1–2 kHz. The fundamental frequency is reflected in the pulse repetition rate and in ripples on the frequency spectrum but not in the number of cycles within a pulse. We suggest that diverticula function as Helmholz absorbers turning the swim bladder into a high-pass filter responsible for the absence of low frequencies typically present in sciaenid calls. Further, we hypothesize that the multicycle pulses are driven by the stretched aponeuroses (flat tendons that connect the sonic muscles to the swim bladder) in this and other sciaenids.

## Introduction

The family Sciaenidae contains 66 genera and almost 300 species^1^. Sciaenids, the subject of important fisheries, are known for their vocalizations leading to the common names croakers or drums. Males in most species emit choruses of croaking or drumming sounds, typically after dusk and sometimes before dawn during the reproductive season^2–6^.

Sciaenids contract sonic muscles that drive swim bladder vibration and sound production^7–9^. Family members have two types of sonic muscles based on their origin and insertion. Extrinsic muscles, the typical condition, originate on a ventral tendon or possibly on lateral muscles, line the edge of the body cavity inside hypaxial trunk muscles and insert on an aponeurosis attached to the dorsal surface of the bladder^4,9–13^. Intrinsic sonic muscles adhere completely to the swim bladder wall^10,13^ and likely represent a derived condition from an extrinsic precursor^14^.

Most sciaenid species exhibit sexual dimorphism with sonic muscles only in males^7,12,13,15^. However, there are several species including *Micropogonias undulatus*, *Micropogonias furnieri*, *Argyrosomus japonicus*, *Larimichthys crocea*, and *Pogonias cromis*^12,16–19^ in which both sexes possess sonic muscles although muscles are larger in male Atlantic croaker^12^. Female sound production in *M. furnieri* and *Pogonias cromis* has been evoked by holding or chasing them^18,19^, and male and female Japanese croaker *Argyrosomus japonicus* and meagre *Argyrosomus regius* produce sounds during the mating season^3,20^. Otherwise female sound production has not been studied in the family.

*Boesemania microlepis* (Boeseman croaker or smallscale croaker), a highly desirable food fish, can grow to over 1 m and weigh 6 kg^21,22^. It is the only described freshwater sciaenid in the Indo-west Pacific area^1^ and one of the few freshwater sciaenid species found outside of the Americas^23,24^. It lives in rivers in Cambodia, Laos, Indonesia, Malaysia, in Vietnam’s Mekong basin, and Thailand. According to an unpublished report by Borsani quoted by Baird^22^, *B. microlepis*’ sounds last about 100 ms and include energy to 6 kHz with a peak frequency of 0.5 kHz. However other frequency and temporal characteristics of the call and sound-producing muscles have not been described, and no formal data have been reported. Staff members at the *B. microlepis* Propagation Station in Thailand stated that the fish starts to call in late afternoon and continues after sunset.

*Boesemania microlepis* has an unusual swim bladder with six long-slender diverticula on each side projecting caudally to the back of the bladder^10,25,26^. The function of these diverticula has not been examined, and we hypothesized that they affect sound spectra. The aims of this study were to describe its swim bladder, sonic muscles and aponeurosis in more detail, its sound characteristics in captivity and in natural waters and determine periods of vocal activity. We find an unusual frequency spectrum that de-emphasizes low frequencies, which we ascribe to the diverticula functioning as Helmholz absorbers.

## Results

**Figure 1:**
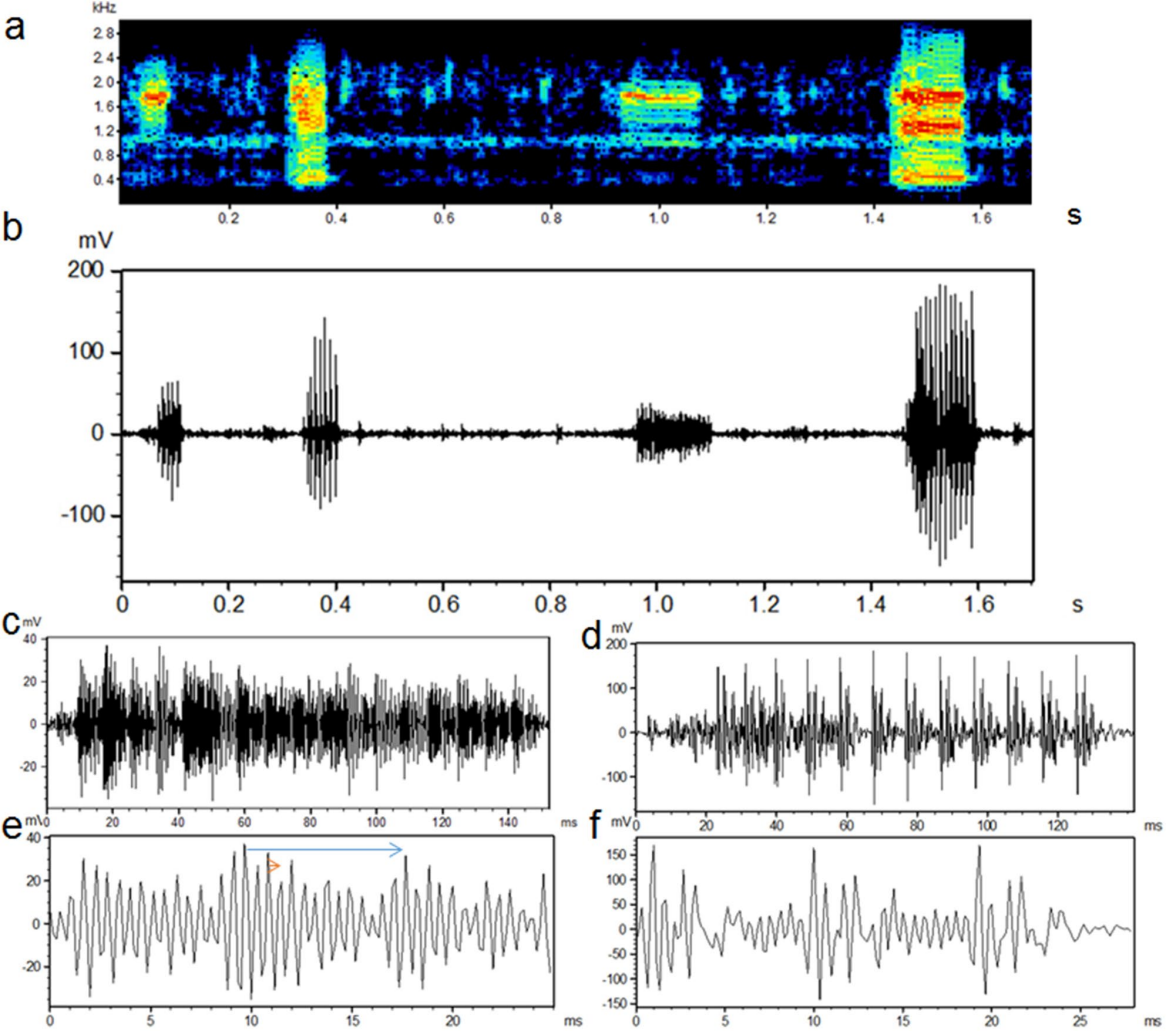
Spectrogram (**a**) and oscillograms (**b**) for 4 captive *Boesemania microlepis* calls recorded in a cement tank. The waveform is expanded for the third (**c**,**e**) and fourth call (**d**,**f**). The pulse-period (blue arrow) and cycle period (short orange arrow) is shown in (**c**). FFT = 128; overlap = 75%; Hamming window.

*Boesemania microlepis* advertisement calls were composed of a series of 6–32 pulses (mean ± SD: 15.1 ± 4.7 pulses) with a repetition rate of 95–123 pps (108.5 ± 6.6 pps) (Table 1, Figs. 1, 3). Pulse duration varied from 3.4 to 9.4 ms (7.3 ± 1.1 ms) and contained 12–16 cycles (14.8 ± 1.1) adding up to a call duration from 53 to 290 ms (139.0 ± 43.8 ms) for each burst of pulses. Number of pulses and call duration were positively correlated (${\hat{\text{y}}}$ = 0.0091X + 0.00205; r^2^ = 0.96). Amplitude of most calls increased from the first to the 3rd–6th pulse (mode was the 4th), after which amplitude stabilized although the final pulse and sometimes the last few pulses decreased in amplitude (Fig. 1).

**Table 1:** Acoustic characteristics for *Boesemania microlepis* advertisement calls from a concrete tank, the Krasieo Reservoir, and the Bang Pakong River, Thailand.

Recording site	Concrete Tank	Krasieo Reservoir	Bang Pakong River
Calls analyzed	62	62	10
Pulses/call (range; mean ± SD)	6–32; 15.1 ± 4.71	7–25; 15.6 ± 4.2	8–14; 12.0 ± 1.6
Call duration (ms; mean ± SD )	53.0–290.0; 138.9 ± 43.8	114.7 ± 37.9	108.2 ± 14.5
Pulse repetition rate (mean ± SD)	108.6 ± 6.6	110.3 ± 0.4	113.4 ± 7.3
Cepstrum spike (ms)	9.6 ± 0.2	9.4 ± 0.3	8.5 ± 0.1
Pulse period (ms; mean ± SD	9.5 ± 0.6	9.2 ± 0.4	8.7 ± 0.2
Pulse duration (ms; mean ± SD)	7.3 ± 1.1	5.3 ± 0.9	6.7 ± 0.5
Cycles/pulse (range; mean ± SD)	12–16; 14.8 ± 1.1	17–20; 19.1 ± 1.1	9–11; 9.7 ± 1.0
Cycle duration (ms; range; mean ± SD)	0.6–0.7; 0.7	0.5- 0.7; 0.5	0.7–1.3; 0.9 ± 0.2
Mean fundamental frequency (Hz) (estimated by the cepstrum spike)	103.9	106.2	117.5
Mean dominant frequency (Hz)	1263	2065	969
Main frequency peaks (Hz)	800–1000	1000–1800	825
	1600–1700	1900–2500	1000
Frequency range (Hz)	390–3840	760–3800	230–2100

**Figure 2:**
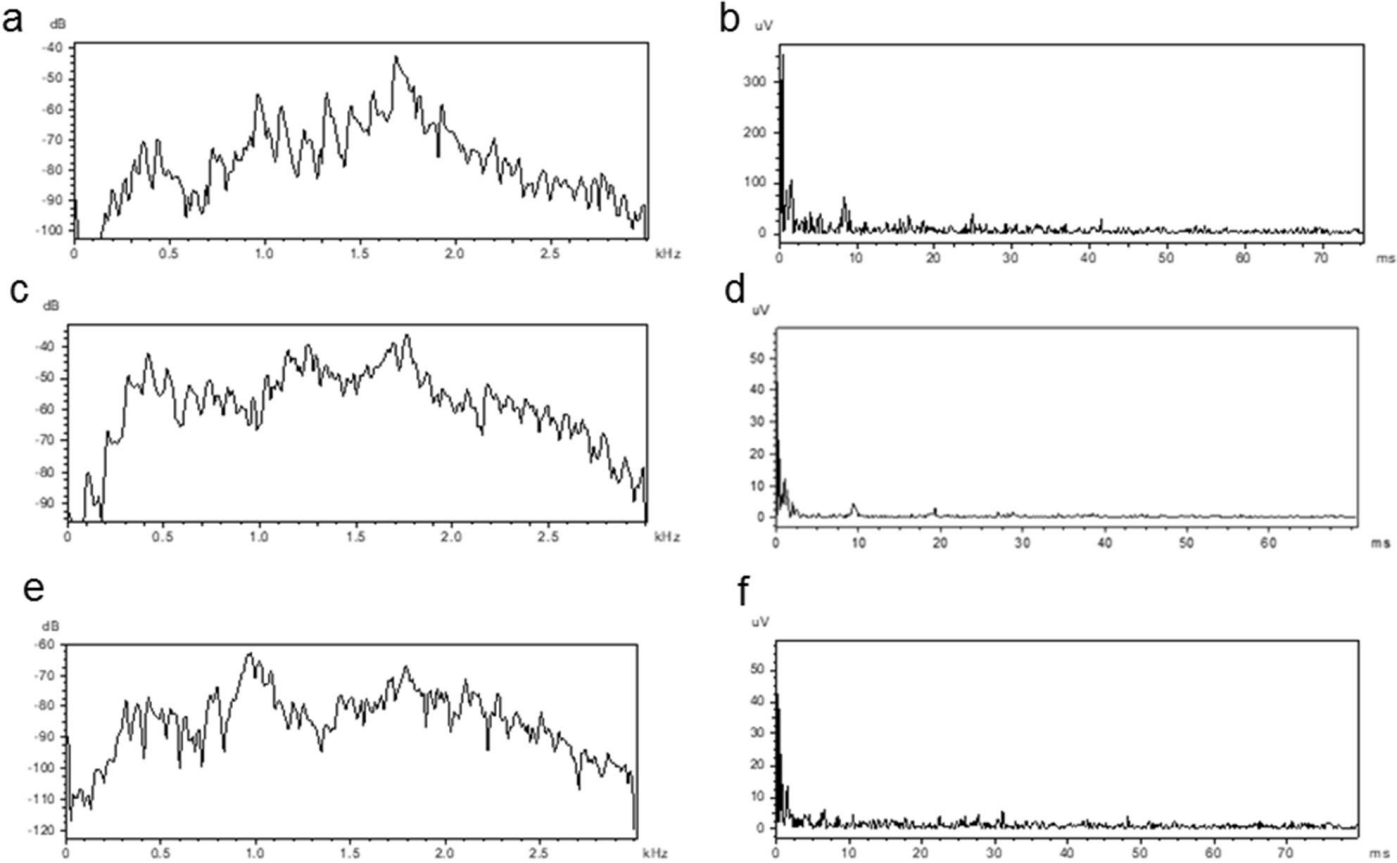
Power spectra (left graphs) and cepstra (right graphs) for the third (**a**,**b**) and fourth call (**c**,**d**) and background noise (**e**,**f**) in Fig. 1.

**Figure 3:**
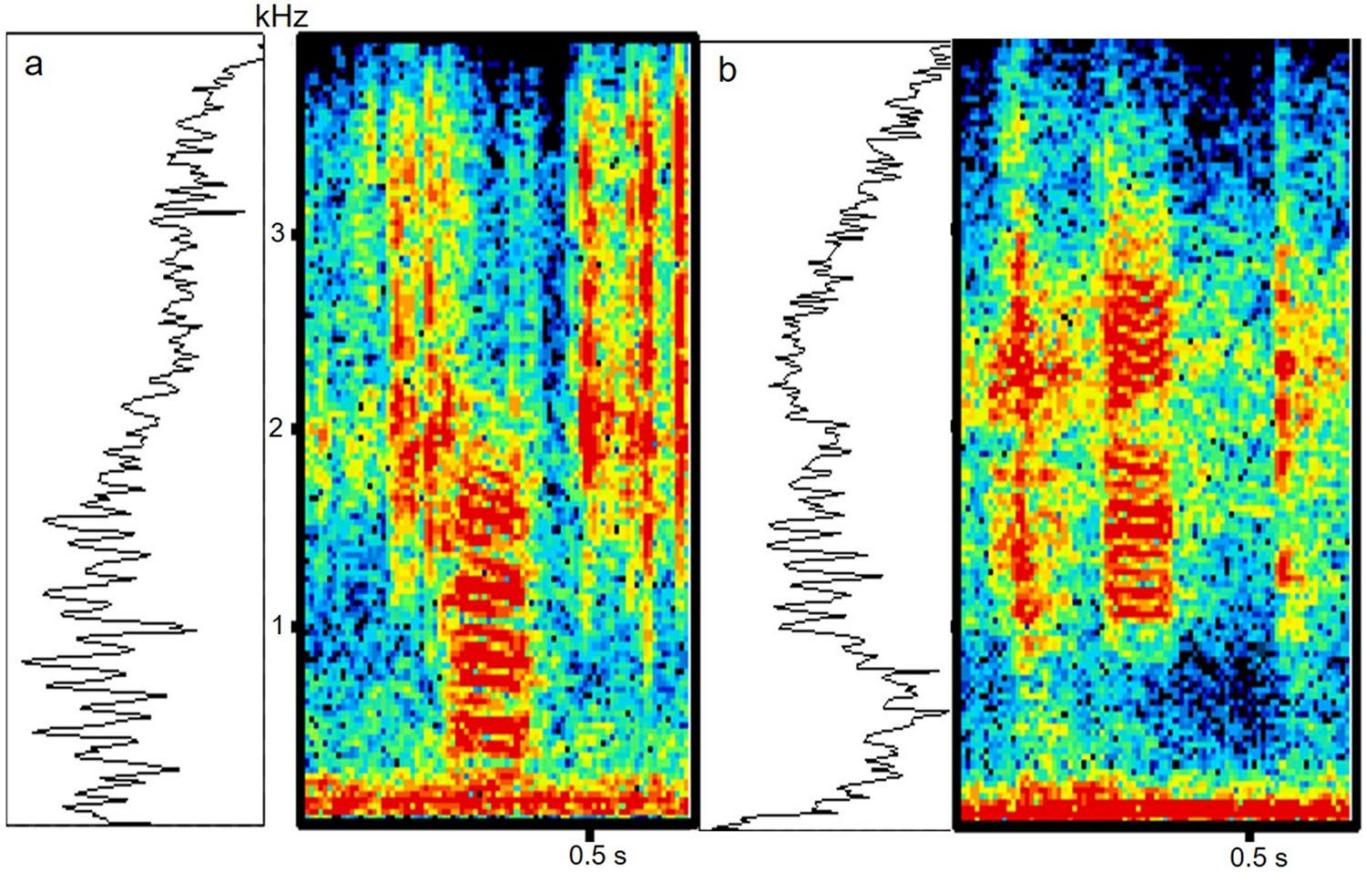
Spectrogram and power spectrum of wild *Boesemania microlepis* tonal calls recorded in the Bang Parkong River (**a**) and Krasieo Reservoir (**b**). Power spectrum FFT = 256; overlap = 75%; Hamming window. The power spectra encompass the tonal sounds and the low-frequency background noise below 0.25 kHz.

Call pulses were tonal with numerous harmonics (Figs. 1, 2, 3). Parameters from the three recording sites were mostly similar but with some differences (Table 1). Unlike typical swim bladder sounds, calls had unusual spectra with decreased amplitudes at low and increased amplitudes at high frequencies. Frequencies of the calls in the tank, reservoir and river ranged from 390–3840, 760–3800, and 230 to 2100 Hz, respectively (Figs. 2, 3). Two main frequency bands occurred in the tank (900–1000 and 1450–1800 Hz) and reservoir calls (1580–1660 and 2000–2500 Hz), and river calls had dominant bands at ca 400–500 and ca. 850 Hz. Cepstrum analysis gave a spike at ca. 9.5 ms for the tank and reservoir calls and 8.5 ms for the river calls, equivalent to an F_0_ of 105 Hz and 118 Hz, respectively (Table 1), which matches the pulse repetition rates of these calls. Numerous small harmonic oscillations occurred at multiples of the fundamental frequency of ca. 105 Hz presented in the power spectra (Figs. 2, 3).

### Nocturnal pattern of sound production

**Figure 4:**
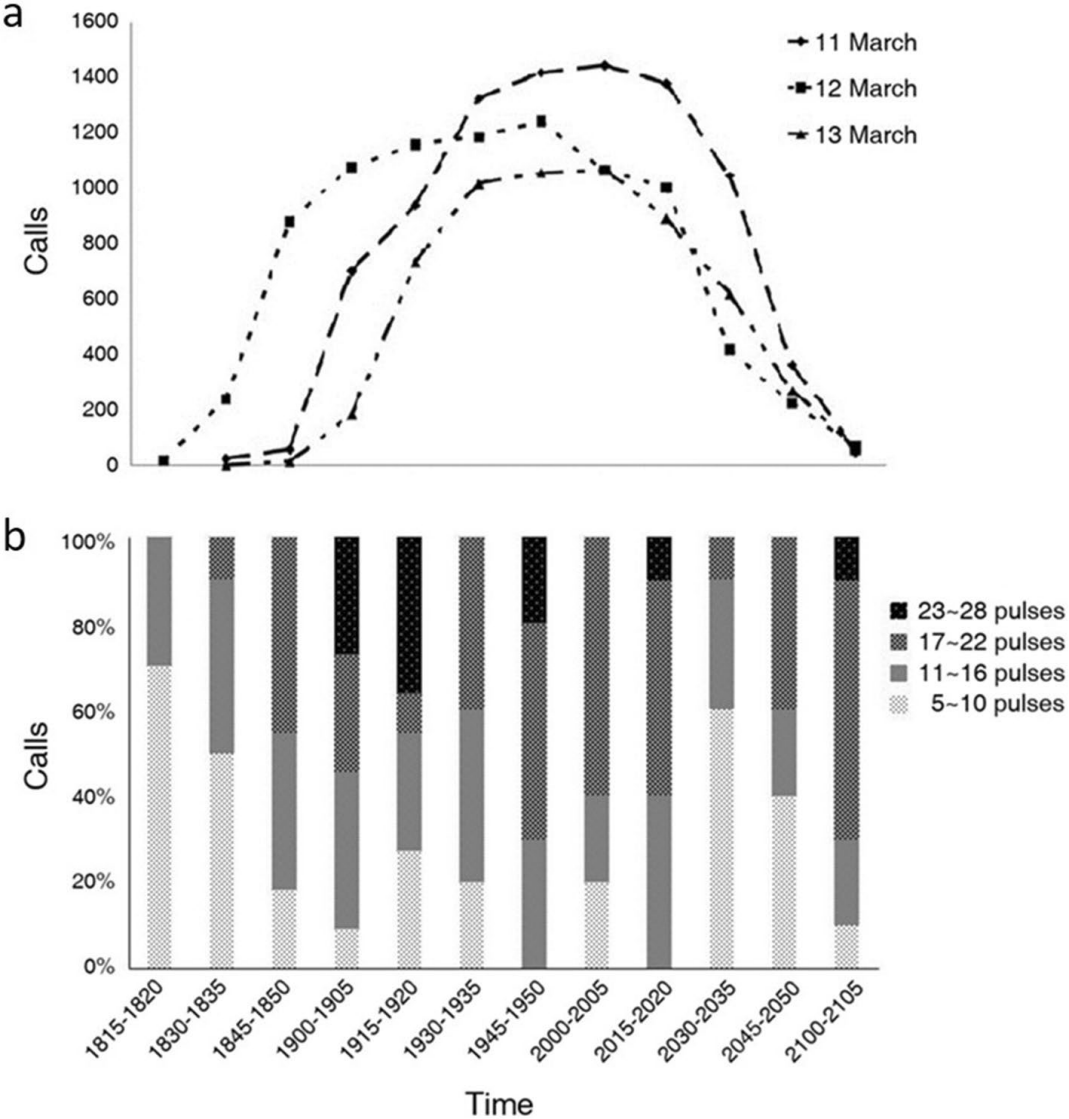
Temporal changes in *Boesemania microlepis* calls. (**a**) number of calls in three consecutive evenings from 11 to 13 March 2011 and (**b**) occurrence of calls with varying pulse numbers in the evening of March 12, 2011.

From 11 to 13 March 2011 vocal activity in the tank began at ca. 1815 hours, peaked between 2000 and 2015 and terminated at ca. 2105 (Fig. 4a). Owing to the small number of fish, calls did not form a continuous chorus, and individual pulses were clear. In the peak period, there were 1072–1447 calls per 5-min. In all three evenings vocal activity started with a few short calls and increased to longer calls (e.g. 23–28 pulses/call) peaking at the end of the first hour (Fig. 4b) before gradually decreasing toward the end of the vocal period.

### Sexual dimorphism of the sonic muscles

**Figure 5:**
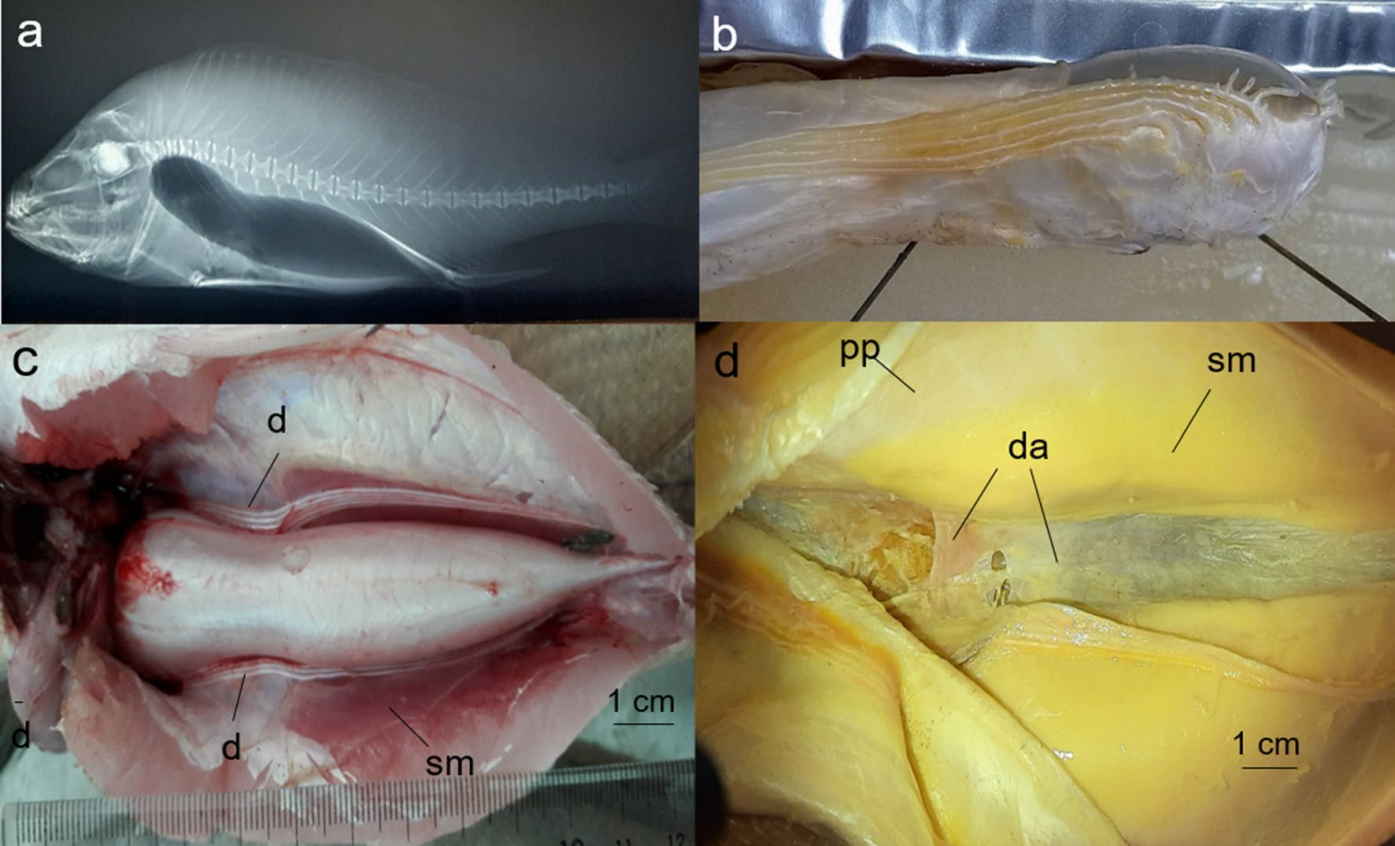
*Boesemania microlepis* swim bladder, diverticula sonic muscles and aponeurosis. (**a**) Radiograph showing the swim bladder. (**b**) Photograph of the swim bladder removed from the fish showing close up of diverticula. (**c**) Photograph of the swim bladder diverticula and sonic muscles. (**d**) Photograph of a fixed specimen with the swim bladder removed showing the dorsal aponeurosis and sonic muscle. The fish had a standard length of = 260 mm and a swim bladder length of 116 mm. The black lines in (**b**) are pins to hold the swim bladder in place. *d* diverticula, *da* dorsal aponeurosis, *pp* parietal peritoneum, *sm* sonic muscle.

The swim bladder is carrot-shaped with a slightly restricted zone at its anterior neck region (Fig. 4). It has a pair of branched cephalic appendages that extend toward the head through the septum transversum^13^. Six additional pairs of fingers (diverticula) originate from the anterior sides and extend caudally ending at the termination of the bladder lumen but before the posterior tip of the swim bladder (Fig. 5a). The anterolateral most finger was shortest. The openings to the diverticula form a relatively straight rostrocaudal line that extends approximately 1.5 cm. Males have a typical red-colored extrinsic sonic muscle surrounding the swim bladder behind the narrow neck (Fig. 5b,c). The sonic muscle attaches to its insertion, a typical aponeurosis above the bladder. However, there is an unusual thickened collar at the anterior edge of the dorsal aponeurosis (Fig. 5d). The muscles curve around the body wall adjacent to hypaxial trunk muscle. The internal side of the trunk muscle is sheathed by the parietal peritoneum separating it from the sonic muscle. The peritoneum extends ventrolaterally on the internal surface of the sonic muscle, becoming a thin ventral aponeurosis that extends across the ventral mid-line of the abdomen. Sonic muscle fascicles originate on the internal surface of the lower parietal peritoneum (ventral aponeurosis) between the sonic muscle and the hypaxial trunk muscle. The origin can be mistakenly assumed to attach to hypaxial muscles ventrolaterally in incompletely-dissected specimens. The ventral aponeurosis thickens in an Asian sciaenid (i.e., *Otolithes ruber*; Mok, personal observation). Females lack sonic muscles and both aponeuroses.

## Discussion

Similar to many sciaenids, *B. microlepis* produces calls in late afternoon into evening^2–4,6,15,27–30^. Calls are composed of a rapid series of pulses with a repetition rate of slightly > 100 pps. Pulses are relatively long (ca 7 ms), tonal and composed of multiple cycles (ca 10–20), each < 1 ms in duration. The pulse repetition rate matches a cepstral spike < 10 ms that is not present in background noise. In weakfish *Cynoscion regalis* emg recordings indicate one muscle contraction per sound pulse^8^. Rapid oscillations of < 1 ms are too fast to be caused by individual muscle contractions, and we therefore suggest that each pulse, but not cycles within a pulse, is driven by an individual contraction of the paired sonic muscles.

Except for peak or dominant frequency, sound parameters were relatively consistent across the three sites. In our experience with fish sounds having multiple peaks, two sounds, even from the same fish, may have adjacent peaks that alternate in terms of the greatest amplitude (Mok and Fine personal observations). Therefore, this metric may not be as definitive, simple or meaningful as it sounds, particularly when different peaks are averaged. The frequency spectrum of *B. microlepis* calls is highly unusual in several respects. Spectra have several peaks at frequencies between < 1 to 2 kHz and an absence of low frequencies that would be expected at multiples of the pulse repetition rate^31,32^. Here low frequency is defined relatively as the fundamental frequency and neighboring harmonics whereas high frequency would refer imprecisely to higher harmonics, including energy between 1–2 kHz. The fundamental frequency and the next three harmonics are absent or attenuated. However, within the broad-frequency band between 1–2 kHz, there are smaller oscillations at ca 100 Hz intervals, apparently harmonics of the missing fundamental frequency. In the oyster toadfish for instance, one of the best understood examples of swim bladder sound generation^31,32^, most energy occurs at the fundamental frequency and the second and third harmonics^29,33,34^, which surprisingly are missing in *B. microlepis*. There are several potential reasons for this unusual spectrum including environmental filtering by shallow water and tank artifacts, potential resonant frequency of the swim bladder, or the unusual swim bladder morphology that could turn the swim bladder into a high-pass filter. These topics will be considered briefly.

### Environmental filtering

As is common, none of the fish in this study were recorded under ideal conditions. Fish in the cement tank were within a maximal distance of 1.5 m from the hydrophone whereas fish in the river and reservoir were at unknown distances and depths in deeper water. However, the hydrophone was close to shore in shallow water that would diminish low frequencies with long wavelengths^35–38^. Still sounds from the three disparate sites all had attenuated low frequencies and a broad frequency representation at higher frequencies (peak frequencies of 1263 Hz, 2065 Hz, and 969 Hz for the tank, reservoir and river respectively). Although calculations for the tank indicate a resonant frequency of 1029 Hz^39^, it is clear that sounds did not exhibit a strong peak at this frequency. Furthermore, recordings of characiform (*Prochilodus argenteus, P. costatus, P. lineatus*) and sciaenid (*Argyrosomus regius, Sciaenops ocellatus *and* Umbrina cirrosa*) fishes in cement tanks with relatively similar dimensions exhibited low frequency energy^6,40^. We therefore conclude that the call spectrum of *B. microlepis* is largely related to energy produced by the fish although it is affected by the environment.

### Resonant frequency of the swim bladder

Historically fish swim bladders were modeled as underwater resonant bubbles whose frequency decreases with size and increases with hydrostatic pressure, e.g. depth^42^. The equation of bubble resonant frequency^41,42^ indicates a resonant frequency of 740 Hz at 0.8 m depth for a 34 cm SL *B. microlepis* with a swim bladder length of 10 cm and radius of 2 cm (corrected for an elongated shape following Weston^42^). The equation does not predict the frequency peaks found in the calls. Bubbles are sharply tuned unlike the broad relatively flat spectrum of the fish’s call, which contains multiple peaks. Although it was recognized that fish sounds and sonar returns from fish with swim bladders were less sharply tuned than from a bubble, early interpretations posited damping by surrounding fish tissue^43,44^. Work in the oyster toadfish suggested a new paradigm, namely that sounds are produced as a forced rather than a resonant response^32^, e.g. the muscle contraction rate sets the fundamental frequency, which explains why a field chorus of boatwhistles produced by multiple differently-sized individuals can have a narrow range of fundamental frequencies on the order of 10 Hz^45^. Toadfish sounds decay rapidly following the final muscle contraction indicating that the bladder is responsible for rapid damping^32^. The swim bladder has a high water content (viscous damping) and multiple layers of collagen and elastin, which serve to rapidly attenuate sounds^46^. Therefore, the expression of resonance of the internal bubble is inhibited. Although weakfish sounds decrease in frequency with fish size, the decrease has been related to a longer contraction time of larger muscles and is therefore still a forced response^8^.

Recently a third possibility has been identified in fish sound production in which sonic muscles are connected to a tendon or bone that attaches to the swim bladder, and the stretched and recoiling tendon forces swim bladder movement^14,47–49^. Such sounds will decrease in frequency with fish and tendon or bone size and have multiple cycles following muscle contraction unlike the simple sonic muscle swim bladder system that decays rapidly.

### Relationship of swim bladder morphology to sound spectrum

The swim bladder of *B microlepis* is carrot-shaped terminating in a skinny ducktail, common in sciaenids^10,26^, but the bladder has two unusual modifications. One is a partial constriction toward the back of the anterior end at the rostral edge of the sonic muscles. The constriction may represent an intermediate condition between a simple swim bladder and one that has divided into two chambers as in silver perch and several other sciaenids^25,50^ and many otophysan fishes including some catfishes, characiforms, and cyprinids^51–53^. The anterior neck region bears a number of projections: a rostral pair heads toward the ears^26^ and six (typically) on each side project in a caudal direction ending near the termination of the swim bladder lumen. Caudal to the anterior region, the sonic muscles terminate on a dorsal aponeurosis connecting to the swim bladder, and they originate on a thickened ventral peritoneum separating the sonic muscle and hypaxial muscles. A ventral connection between the sonic muscles has been mentioned for weakfish *Cynoscion regalis*^11^ and Atlantic croaker *Micropogonias undulatus*^9^. In Atlantic croaker, material properties (stress at break and Young’s modulus) are greater in the aponeurosis than in bladder tissue although strain is similar^54^ suggesting that the aponeuroses will be stretched by the sonic muscle, acting like a tendon, and transfer muscle strain energy back to the swim bladder. Based on geometry, the middle to back part of the bladder will be compressed during muscle contraction forcing gas into the anterior region, which will expand. The stretched tendon likely explains the multiple cycles caused by a single muscle contraction^55^.

### Diverticula

There has been almost no experimental work on the various diverticula of sciaenids and no insight as to their ancestral function or how they might have evolved. Locascio and Mann^56^ suggested that multiple tubercles on the swim bladder in black drum *Pogonias cromis* increase the bladder’s surface area, which would increase volume velocity and therefore sound amplitude^32,57^. Yan and Fine (unpublished) examined hearing in four Atlantic sciaenids with a range in diverticula length: long terminating adjacent to the ears (sliver perch and weakfish), medium (typically extending to the pericardium in Atlantic croaker) and minimal (spot). Deflation of swim bladder gas via a syringe caused a > 10 dB decrease in auditory thresholds in silver perch and weakfish but had no effect in croaker or spot. Therefore, the swim bladder only aids hearing when diverticula terminate close to the ears^58,59^. As with silver perch which can hear to 4 kHz^50^, it is likely that *B. microlepis*, with two long anterior diverticula^26^, has sensitive high-frequency hearing, which would match the elevated frequency distribution of its sounds. Such elevation would increase their communication frequency band above much of background noise caused by waves and bubbles and sounds produced by other nonspecialized fishes, e.g., a relatively unobstructed sound channel.

The waveform of black drum advertisement calls, a sciaenid^18,56,60^, shares similarities with the toadfish boatwhistle^32^: both produce a long tonal call in which each contraction causes a sound cycle, and the sound attenuates rapidly following the final contraction. Both species have intrinsic muscles, and both however are unlike typical sciaenid calls that have a more complex waveform. For instance, the weakfish sound pulse has at least two cycles for each muscle action potential (emg)^8,61^. A number of sciaenids produce multiple cycles per sound pulse—considerably more than two—including *Sciaenia umbra*^62^, *Cynoscion guatucupa*^63^, *Aplidinotus grunniens*^64^, and *Umbrina canosai*^65^. Therefore, of the three potential mechanisms of swim bladder sounds (resonant bladder, forced response determined by sonic muscles or forced response determined by muscles with an intermediate tendon or bone), sciaenid sounds are likely caused by the third mechanism, e.g. multiple cycles determined by a combination of the dorsal and ventral aponeurosis continuing to excite the bladder. This statement requires experimental verification, but with current knowledge, it is a parsimonious interpretation.

### Absence of low frequencies in the call spectrum

We hypothesize that the multiple side appendages turn the swim bladder into a high-pass filter that will function as a Helmholz absorber^66,67^, a concept unexplored in fish bioacoustics. Tang^66^ notes Helmholtz resonators are widely used as silencing devices in ducted systems because of their strong sound attenuation and have been useful in dealing with the low-frequency noise propagating inside air conditioning ductworks. We suggest that that pressure from the gas being pushed into the long slender diverticula of the swim bladder will be dissipated by friction at low frequencies and favored at higher frequencies. The lateral most diverticulum is notably shorter than the others, which also have different lengths depending on their rostrocaudal position as they exit the bladder. Therefore, the different diverticula may be tuned to somewhat different frequencies. Additionally, we are unaware of how soft pliable material capable of viscous damping^46^ will affect the system. Our hypothesis clearly requires experimental evidence for confirmation.

### Species distribution

*Boesemania microlepis* lives primarily in freshwater^22^ but may also occur in estuaries^68^. Conversely, *Aspericorvina jubata, another sciaenid,* occurs in shallow coastal waters and occasionally enters estuaries and rivers^68^. Therefore, *B. microlepis* and *A. jubata* are the only identified sciaenid species that can possibly be found in the rivers connected to the Gulf of Thailand. Whether the former can extended into the upstream section of the Bang Pakong River remains unclear. A possible new sciaenid species called the golden croaker by local fishers has been captured in the lower Bang Pakong River. However, *B. microlepis* is the only known sciaenid in the tank, reservoir and the upstream part of the Bang Pakong River.

## Methods

### Ethical considerations

Observations in this investigation were done in compliance with the ethical rules of the IACUC of National Sun Yat-sen University. The only interaction with living animals was placing a hydrophone in the water; thus no permits were required.

Small scale croakers, *B. microlepis*, were recorded from captive fish raised at the Freshwater Fisheries Research and Development Centers in Chainat, Thailand, and from wild fish in the Krasieo Reservoir and the Bang Pakong River, Thailand. Recordings in 2011 used an HP-A1 hydrophone (Burns Electronics; frequency range from 10–25,000 Hz ± 3 dB) and a HP-A1 Mixer-amplifier connected to a Korg digital recorder (MR¬1000, 44.1 kHz with 16-bit resolution), and 2019 recordings used a H2A hydrophone (Aquarian Audio Products; useful frequency range: < 10 Hz to > 100 kHz) and Sony digital recorder (PCM-M10). For field recordings the hydrophone was attached to a floating platform anchored from shore (suspended 1 m from the surface at a water depth ca 1.5 m). Water temperatures were 29.8 °C in the Bang Pakong River, 28.2 °C in the Krasio Reservoir and 28.2 in the Chaopraya River close to the Chainat Fisheries Station.

Twelve captive fish (average total length ca. 40 cm) from the fisheries center were recorded in a 3 × 2 × 0.8 m concrete tank filled with freshwater to a 0.7 m depth between March 11 to 13, 2011 The hydrophone was placed in the center of the concrete tank about 45 cm beneath the surface. Following the advice of staff members at the Research and Development Center, recording started at 1600 hours and stopped when no calls were produced for 15 min.

Wild *B. microlepis* have been confirmed at Krasieo Reservoir located in Dan Chang, Supanburi Province, Thailand (S. Sirisuary personal observation). A possible new *Boesemania* species lives in the Bang Pakong River in the downstream and estuarine parts of the river), but sound recordings were made at upstream sites in November 2012 where only *B. microlepis* is known. Krasieo reservoir sounds were recorded from 1600 to 2100 hours, 19, Nov. 2019. Recordings in the Bang Pakong River were made on 3 and 4 November 2012 between 1700 and 1900 hours under a bridge beside the riverbank (near Wat Khao Din, Chon Buri Chaehoengsao).

### Sound analysis

Ten tank calls with good signal to noise ratios were chosen from 5 min sections every 10 min for the three nights. Sounds were analyzed using Raven Pro 1.4 (Bioacoustics Research Program, The Cornell Lab of Ornithology, Ithaca, NY, USA; Charif et al. 2010) and Avisoft-SASLab Pro 5.2.08. We measured call duration (ms), number of pulses per call, pulse repetition rate, inter-pulse interval (IPI, measured from the end of one pulse to the beginning of the next, ms), pulse duration (measured as the time between the beginning to the end of one pulse, ms), and major frequency bands. Data are presented as means ± 1 standard deviation. Frequency parameters were measured from sonograms and temporal features from oscillograms. Cepstral analysis was conducted to reveal call fundamental frequency. In cepstral analysis, an inverse Fast Fourier Transform is applied to a log power spectrum. For a harmonic sound, a sharp spike will be displayed at around T seconds, the period of the fundamental frequency, calculated by F_0_ = 1/T. (https://www.phon.ox.ac.uk/jcoleman/new_SLP/Lecture_7/Cepstral_analysis.html).

### Sonic apparatus

Twenty fish (average standard length 40.3 cm; 11 females and 9 males) were purchased from local fishmongers at Sam Chuk Old Market in Suphan Buri Province, Sam Chuk District on July 9th in 2011 and Nov. 28, 2012. Sex was determined by appearance of the gonads, and gross anatomy of the swim bladder, sonic muscles and aponeuroses was described.
